# Supplementary material for: Venomix: a simple bioinformatic pipeline for identifying and characterizing toxin gene candidates from transcriptomic data
Source: PeerJ. 2018 Jul 31;6:e5361. doi: 10.7717/peerj.5361 (PMC6074769; doi:10.7717/peerj.5361)
Supplement: Supplemental Information 6 [file peerj-06-5361-s006.gz › FinalOutput_1E-6/Gigantoxin-1_2/finaltree.pdf]

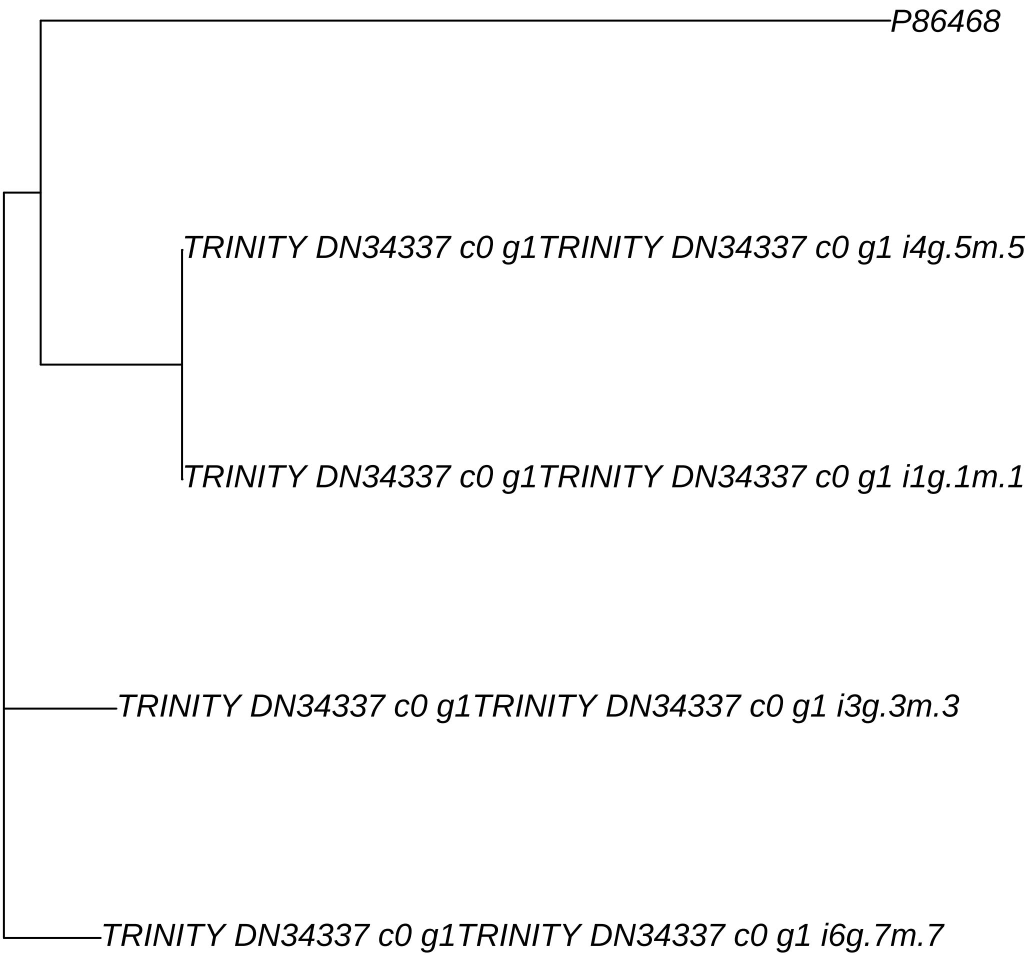
P86468
TRINITY DN34337 c0 g1TRINITY DN34337 c0 g1 i4g.5m.5
TRINITY DN34337 c0 g1TRINITY DN34337 c0 g1 i1g.1m.1
TRINITY DN34337 c0 g1TRINITY DN34337 c0 g1 i3g.3m.3
TRINITY DN34337 c0 g1TRINITY DN34337 c0 g1 i6g.7m.7
